# The ABC of non-inferiority margin setting: an investigation of approaches

**DOI:** 10.1186/1745-6215-12-S1-A34

**Published:** 2011-12-13

**Authors:** Steven A Julious

## Objectives

To assess the efficacy of a new investigative treatment a non-inferiority study is undertaken when it is no longer ethical to have a placebo control. Instead an active controlled trial is undertaken. The objective is thus to show that the new treatment is no worse than the active control.

In analysing a non-inferiority trial, the following ABC needs to be considered [1]:

1. The **A**ssay sensitivity of the active control in both the placebo controlled trials and in the active controlled non-inferiority trial is the same.

3. **B**ias is minimised through steps such as ensuring that the patient population and the primary efficacy endpoint are essentially the same for the placebo-controlled trial and the active-controlled trial.

2. **C**onstancy assumption of the effect of the common comparator. Such that for two trials in sequence: Trial 1 and Trial 2 the control effect of Treatment B vs. Placebo in Trial 1 is assumed to be the same as the control effect of Treatment B vs. ‘Placebo’ in Trial 2

This presentation will describe how this ABC can be considered.

## **Methods**

A major issue in designing a non-inferiority study is the setting of the non-inferiority limit. The Food and Drug Administration (FDA) discuss setting a limit so it would be possible to demonstrate superiority over placebo. This comparison would need to be done indirectly as placebo is not given concurrently. A margin could be set for non-inferiority therefore which will enable superiority to placebo to be demonstrated

The European regulators discuss surveying experts to quantify the margin. An approach that uses both the objective observed data and subjective opinion to set a non-inferiority margin would useful which lends to Bayesian approaches.

## Results

There is an issue with indirect comparisons if they are done retrospectively as the effect over placebo may not be as great today as when a placebo controlled trial was last undertaken [2]. The presentation will give a number of examples where the effect of treatment has fallen over time. This is known as placebo creep and could bias the estimate of effect over placebo. Comparison will be made of simple indirect and Bayesian approaches to set non-inferiority margins

## Conclusions

When making indirect comparison over time to determine a non-inferiority margin, if there is a suspicion of placebo creep then the simple ABC for setting a margin may fail and approaches such as a simple Bayesian approach may need to be considered.

## References

[B1] Julious SA The ABC of non-inferiority margin setting from indirect comparisons Pharmaceutical Statistics 2011 DOI: 10.1002/pst.517 10.1002/pst.517 21928354

[B2] Julious SA Wang SJ Issues with indirect comparisons in clinical trials particularly with respect to non-inferiority trials DIJ 2008 42 6 625 33

